# Gut microbiota transmits osteoimmune dysregulation and exacerbates trabecular bone loss in tristetraprolin-deficient mice

**DOI:** 10.1007/s00774-026-01753-9

**Published:** 2026-08-17

**Authors:** Kyu Hwan Kwack, Jiho Sohn, Lixia Zhang, Lu Li, Reegan Mullaney, Yukitomo Arao, Perry J. Blackshear, Keith L. Kirkwood

**Affiliations:** 1 Department of Oral Microbiology, College of Dentistry, Kyung Hee University, Seoul, South Korea; 2 Department of Oral Biology, State University of New York at Buffalo, University at Buffalo, Buffalo, NY USA; 3 Division of Digestive and Liver DiseasesDepartment of Medicine, University of Texas Southwestern Medical Center, Dallas, TX USA; 4 Molecular and Cellular Biology Laboratory, National Institute of Environmental Health Sciences, Research Triangle Park, Durham, NC USA; 5 Departments of Biochemistry & Medicine, Duke University Medical Center, Durham, NC USA; 6 Department of Oral Biology, School of Dental Medicine, University at Buffalo, 645 Biomedical Research Building, 3435 Main St, Buffalo, NY USA

**Keywords:** Tristetraprolin, Gut microbiota, Sterile inflammation, Osteoimmunology, Monocytic myeloid-derived suppressor cells

## Abstract

**Introduction:**

Tristetraprolin (TTP) is an RNA-binding protein essential for controlling cytokine production, and its deficiency leads to profound skeletal deterioration. Although TTP deficiency is associated with systemic inflammation and microbial dysbiosis, the contribution of the gut microbiota to bone pathology remains poorly defined.

**Materials and methods:**

We investigated whether the microbiome causally modulates osteoimmune mechanisms and bone microarchitecture in TTP-deficient mice. To isolate the effects of the microbiome, we utilized specific pathogen-free (SPF) and germ-free (GF) co-housing mouse models.

**Results:**

Microbial transfer bidirectionally regulated systemic inflammation and the expansion of monocytic myeloid-derived suppressor cells (M-MDSCs), a population with potent osteoclastogenic capacity. Importantly, microbiota transfer was sufficient to induce osteoclast activation and a selective deterioration of trabecular bone microarchitecture in otherwise healthy mice, without affecting overall bone mass. Crucially, the transmission of these osteoimmune and skeletal phenotypes was microbiota-dependent; while a baseline genetic bone deficit persisted in GF TTP-deficient mice, the co-housing-induced M-MDSC expansion and trabecular bone alterations were not observed under GF conditions.

**Conclusion:**

Our findings identify a microbiota-dependent osteoimmune axis that amplifies inflammatory bone loss in TTP deficiency. This work establishes the gut microbiome as a mechanistic modifier of bone quality in genetically driven inflammatory disease and highlights microbial targeting as a potential therapeutic strategy for inflammatory bone loss.

**Supplementary Information:**

The online version contains supplementary material available at https://doi.org/10.1007/s00774-026-01753-9.

## Introduction

Systemic chronic inflammation is a fundamental driver of numerous chronic diseases, including cardiovascular disease, autoimmune disorders, and osteoporosis [1]. A low-grade, persistent inflammatory state associated with aging, termed ‘inflammaging’, is a key mechanism that promotes immunometabolic dysfunction and skeletal tissue damage [2, 3]. This chronic inflammation disrupts bone homeostasis, leading to reduced bone density and a deterioration of its microarchitecture [4]. Therefore, understanding the fundamental regulatory mechanisms of inflammaging is crucial for promoting musculoskeletal health.

Tristetraprolin (TTP) is a crucial RNA-binding protein that maintains immune and skeletal homeostasis by regulating inflammatory mediators like tumor necrosis factor-alpha (TNF-α) [5–8]. Critically, TTP expression declines with age, suggesting that TTP-deficient (TTPKO) mice are a highly relevant model for studying inflammaging [9]. TTPKO mice exhibit severe, systemic sterile inflammation, autoimmunity, and a cachexia-like phenotype [8, 10]. Our previous studies have demonstrated that TTP deficiency leads to a dramatic expansion of monocytic myeloid-derived suppressor cells (M-MDSCs), which serve as potent osteoclast precursors that enhance bone resorption, contributing to severe bone loss [9, 11]. This phenotype, characterized by chronic inflammation and dysregulated immune function, represents an accelerated form of immunosenescence.

Inflammaging and immune dysfunction are deeply intertwined with the gut microbiota, which is known to influence skeletal homeostasis through systemic immune modulation [12–14]. We previously found that TTP-deficient mice exhibit significant alterations in gut microbiota composition that become more pronounced as the inflammatory phenotype progresses [10]. Furthermore, the observation that antibiotic treatment mitigates systemic inflammation in these mice strongly implicated the microbiota as a key modifier of the pathology [15].

In this study, we test the hypothesis that the dysbiotic microbiota in TTP-deficient mice is a transmissible factor that actively exacerbates the host’s genetic predisposition to inflammation and skeletal fragility. We posit that the host’s genetic deficiency (TTPKO) initiates a vicious inflammatory feedback loop that is amplified by the microbiome. Using co-housing and germ-free (GF) mouse models, we aimed to determine the role of the gut microbiota in modulating and transmitting TTP-mediated systemic sterile inflammation, M-MDSC expansion, and associated trabecular bone deterioration.

## Materials and methods

### Mice and housing

All mice used in this study were on a C57BL/6 genetic background and obtained through a Material Transfer Agreement with P. Blackshear, National Institute of Environmental Health Sciences (NIEHS). Specific pathogen-free (SPF) wild-type (WT) and TTP-deficient (TTPKO) mice were generated through in-house breeding and maintained under SPF conditions. Germ-free (GF) mice were bred and housed at National Institute of Environmental Health Sciences (NIEHS) gnotobiotic facility. The aged-matched (30 weeks old) GF mice were shipped under sterile conditions and were euthanized for experiments immediately upon arrival. Genotyping for all mice was performed by PCR analysis of genomic DNA. All experiments were performed in accordance with relevant guidelines and regulations. This study was approved by the University at Buffalo’s Institutional Animal Care and Use Committee (protocol #ORB15087Y). All animal protocols were conducted with strict adherence to standards articulated in the Animal Research: Reporting of In Vivo Experiments (ARRIVE) guidelines. All mice were maintained under a 12-h light and 12-h dark cycle and provided ad libitum access to chow. Mice (weights approximately 20–25 g) were euthanized using carbon dioxide (CO₂) inhalation in accordance with the AVMA Guidelines for the Euthanasia of Animals (2020) and institutional IACUC-approved protocols.

### Co-housing experiment

To investigate the influence of gut microbiota on TTP-mediated inflammation, a co-housing model was established immediately after weaning. This model leveraged the natural coprophagic behavior of mice to facilitate the bidirectional exchange of microbial communities. Mice were grouped as follows: WT mice housed with WT mice (WT), TTPKO mice housed with TTPKO mice (TTPKO), WT mice housed with TTPKO mice (WT-CH), and TTPKO mice housed with WT mice (TTPKO-CH). Mice were allocated to experimental groups (WT, TTPKO, WT-CH, TTPKO-CH) by simple randomization at weaning to minimize allocation bias. No animals or data points were excluded from analyses unless predetermined quality-control criteria for sequencing or histology were not met. All inclusion/exclusion criteria were established a priori. This study exclusively used female mice to evaluate the transmissible osteoclastogenic and skeletal phenotypes associated with TTP deficiency. We acknowledge that sex is an important biological variable in both bone metabolism and immune regulation. Future studies incorporating male animals will be necessary to determine whether the microbiota-dependent osteoimmune axis described here operates in a sex-dependent manner.

### Serum enzyme-linked immunosorbent assay

Following euthanasia, blood samples for serum were obtained from each animal via cardiac puncture. The collected blood was allowed to incubate at room temperature for 30 min and then centrifuged at 1000 × g for 10 min to extract the serum. The serum levels of TNF-α were measured using a microplate reader (BioTek Instruments, Winooski, VT, USA) following the manufacturer’s instructions (R&D Systems, Minneapolis, MN, USA).

### Microbiome sequencing and analysis

DNA was extracted from fecal and cecal samples using the DNeasy PowerSoil Kit (Qiagen, Hilden, Germany). 16S rRNA gene amplicon libraries targeting the V3-V4 hypervariable region were prepared and sequenced, following protocols described in a previous study [16]. Raw sequences were processed using Mothur (v1.44.3). Specifically, paired-end reads were first joined into contigs, and those containing ambiguous bases, shorter than 150 bp, or with more than 10 homopolymers were excluded. Chimeric sequences were identified and removed using UCHIME. Thus, a total of 4,313,988 sequences were retained (mean ± SD per sample = 51,357 ± 14,629) for downstream analysis. Individual sequences were classified using the Ribosomal Database Project (RDP) classifier in Mothur, with a custom reference database combining the Genome Taxonomy Database (GTDB v202) and the Human Oral Microbiome Database (HOMD v15.22) to improve species-level resolution and annotation completeness as described [17]. Reads were clustered into operational taxonomic units (OTUs) at 97% similarity. OTU taxonomy was assigned using Mothur’s *classify.otu* command with an 80% consensus confidence threshold. To improve species-level identification, representative sequences from OTUs unclassified at the species level were compared against the NCBI-curated 16S rRNA database using BLAST. Top matches (presenting at least 97% similarity and coverage) were used to provide additional species-level taxonomy information for each OTU.

Alpha diversity was assessed using the Shannon index after rarefying to 20,208 reads per sample (the minimum observed across samples), repeated 1000 times with the average reported. Beta diversity was evaluated using Bray–Curtis distances calculated from the OTU relative abundance matrix and visualized via principal coordinate analysis (PCoA). Relative abundance data were normalized using a centered log_2_-ratio (CLR) transformation to account for the compositional structure of the data and reduce the likelihood of spurious correlations. Linear discriminant analysis effect size (LEfSe) was used to identify differentially abundant species across groups. To quantify microbial compositional shifts, we developed a dysbiosis score that captures the deviation of the cecal microbiome from WT toward the TTPKO profile. For each sample, the dysbiosis score was calculated as follows: Dysbiosis Score = mean CLR abundance of species enriched in TTPKO − mean CLR abundance of species enriched in WT. This metric was applied across all groups (WT, WT-CH, TTPKO-CH, TTPKO) to evaluate the extent of microbiome deviation from the reference state.

### Histology

Small intestine and colon samples were collected and fixed in formalin for 24 h. The formalin-fixed samples were embedded in paraffin and sectioned horizontally into 5 µm-thick sections. Subsequently, the samples were stained with hematoxylin and eosin. The histology score was calculated based on inflammation severity and extent, epithelial cell changes, and mucosal architecture [18].

### Isolation of tissue cells and flow cytometry

Bone marrow cells were obtained from the femur, while splenocytes were acquired by mincing the spleen. Erythrocytes were lysed using ACK lysis buffer (Gibco, Grand Island, NY, USA), and the cell suspension was passed through a 70 µm strainer (Corning, New York, NY, USA) to obtain a single-cell suspension. The cells were pre-treated with CD16/32 Fc block (BD, BD Biosciences, San Jose, CA, USA) and then stained with a viability dye and antibodies against CD11b, Ly6C, Ly6G, and F4/80 (Miltenyi Biotec, Bergisch Gladbach, Germany). Cells were acquired on an LSR Fortessa flow cytometry (BD Biosciences) and analyzed with FlowJo software (version 10.10). For high-dimensional visualization of myeloid populations, viable CD11b^+^ cells were downsampled, and dimensionality reduction was performed using the PHATE (Potential of Heat-diffusion for Affinity-based Trajectory Embedding) algorithm via FlowJo plugins.

### Micro-computed tomography and analysis

The tibia from each animal was fixed in 10% phosphate-buffered formalin for 24 h and then washed with phosphate-buffered saline. Changes in bone structure were assessed using a Scanco Medical 100µCT scanner (Scanco Medical, Bruettisellen, Switzerland), and three-dimensional images were analyzed using AnalyzePro software (AnalyzeDirect, Inc., Overland Park, KS, USA). Regions of interest were defined according to the methods used in a previous study [11].

### Tartrate-resistant acid phosphatase (TRAP) staining and histomorphometry

Following micro-CT scanning, the fixed tibiae were decalcified in 0.5 M EDTA at pH 8.0 for 14 days and then embedded in paraffin. Sagittal sections of 5 µm thickness were prepared and stained for tartrate-resistant acid phosphatase (TRAP) to identify multinucleated osteoclasts; counterstaining was performed with Fast Green and hematoxylin following standard protocols. Images were captured using a bright-field microscope. Osteoclast parameters, including the number of TRAP-positive osteoclasts per bone perimeter (N.Oc/B.Pm,/mm) and osteoclast surface per bone surface (Oc.S/BS, %), were quantified in the secondary spongiosa of the proximal tibia using ImageJ software in a blinded manner. The parameters were measured in accordance with the American Society for Bone and Mineral Research (ASBMR) histomorphometry guidelines [19].

### Statistical analysis

Sample sizes were based on prior studies using similar models and outcomes. Investigators conducting histological scoring, micro-CT analysis, and flow cytometry analysis were blinded to group identity during data acquisition and analysis. Both male and female mice were included in breeding colonies; however, experimental groups consisted exclusively of female animals to reduce confounding. Exact sample sizes (n) for each experiment are detailed in the respective figure legends. Occasional variations in the n number between specific downstream assays (e.g., whole-body weights vs serum ELISA vs. histology) within the same experimental cohort are due to limited sample volume or exclusions based on predefined assay quality metrics. Statistical analysis was conducted using GraphPad Prism 10.4 (GraphPad Software Inc., La Jolla, CA, USA), employing unpaired Student’s t-test and one- and two-way analysis of variance (ANOVA) followed by Tukey’s multiple comparisons test.

## Results

### Co-housing remodels the gut microbiota and normalizes dysbiosis in TTP-deficient mice

**Fig. 1:**
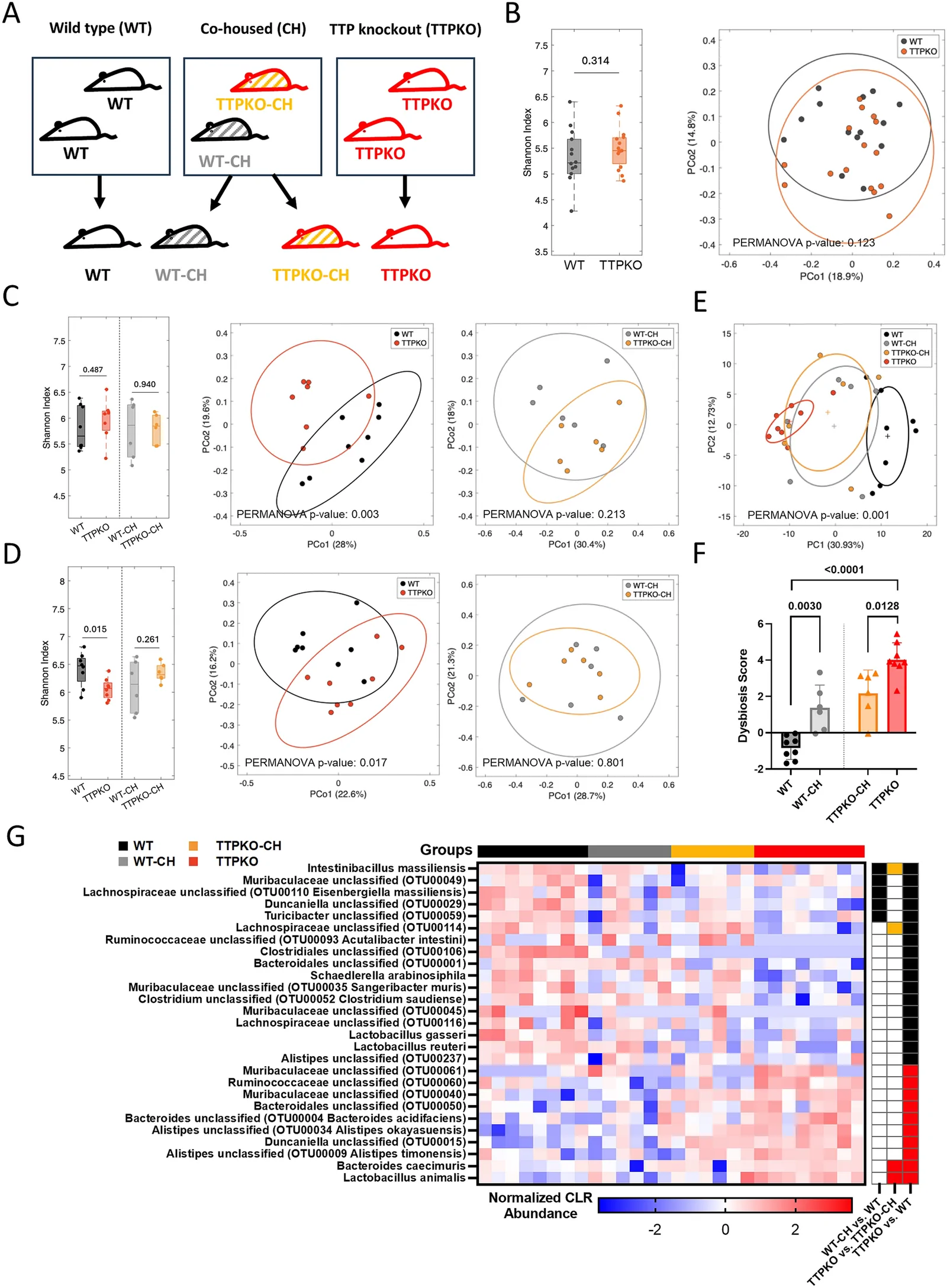
Co-housing remodels the gut microbiota and normalizes dysbiosis in TTP-deficient mice. **A** Schematic of the experimental design for co-housing. Wild-type (WT) and TTP-knockout (TTPKO) mice were housed together (WT-CH and TTPKO-CH) or separately (WT and TTPKO) from 3 weeks of age. **B** Alpha diversity (Shannon index) and beta diversity (PCoA of Bray–Curtis dissimilarity) of the gut microbiota at the 3-week baseline. **C** Alpha and beta diversity of fecal microbiota at 18 weeks of age. **D** Alpha and beta diversity of cecal microbiota at 33 weeks of age. **E** PCoA plot of beta diversity showing all four experimental groups at 33 weeks. **F** Microbial dysbiosis score calculated for each group at 33 weeks. **G** Heatmap showing the centered log-ratio (CLR) abundance of operational taxonomic units (OTUs) normalized to Z-scores at week 33. OTUs were identified as differentially abundant based on LEfSe analysis with an LDA score ≥ 3 comparing WT and TTPKO groups. The color bar indicates the enrichment of each OTU in WT-CH vs. WT, TTPKO vs. TTPKO-CH, and WT vs. TTPKO, respectively, as determined by LEfSe. For the complete list using an LDA score cutoff of 2, see Supplementary Fig. 3. Statistical significance was determined by Student’s t-test, one-way analysis of variance with Tukey’s multiple comparisons test or PERMANOVA for beta diversity. Data are represented as mean ± SEM (n = 8 for WT, n = 6 for WT-CH, n = 6 for TTPKO-CH, n = 8 for TTPKO)

To determine if the gut microbiota is a transmissible factor in TTP-mediated inflammation, we co-housed wild-type (WT) and TTP-knockout (TTPKO) mice starting at 3 weeks of age and monitored their gut microbial composition over time (Fig. 1A). At the 3-week baseline, there were no significant differences in fecal microbiota between WT and TTPKO mice in either alpha or beta diversity (Fig. 1B). However, as the inflammatory phenotype progressed, differences in fecal beta diversity became apparent by 18 weeks (Fig. 1C), and by 33 weeks, analysis of cecal microbiota revealed significant differences in both beta and alpha diversity (Fig. 1D). To identify taxa distinguishing WT and TTPKO mice, we performed Linear Discriminant Analysis Effect Size (LEfSe) analysis on cecal microbiota at week 33 (Supplementary Fig. 1). TTPKO mice exhibited a lower abundance of commensal families such as *Muribaculaceae* and *Lachnospiraceae,* and a higher abundance of species such as *Bacteroides caecimuris,* which has been previously associated with inflammation in murine colitis models [20]. Co-housing altered these trajectories by inducing convergence of the microbial communities. Principal Coordinates Analysis (PCoA) of Bray–Curtis beta diversity showed that the distinct profiles of WT and TTPKO mice shifted toward each other when co-housed, with WT-CH and TTPKO-CH groups clustering more closely than their non-co-housed counterparts (Fig. 1E). To further quantify these changes, we applied a microbial dysbiosis score. Dysbiosis scores in the co-housed groups (WT-CH and TTPKO-CH) were intermediate between WT and TTPKO, supporting a bidirectional exchange in which WT-CH mice shifted toward a TTPKO-like profile, while TTPKO-CH mice shifted toward a WT-like profile (Fig. 1F). As shown in Fig. 1G and Supplementary Figs. 2–3, co-housing remodeled the community structure such that TTPKO-CH mice showed restoration of several WT-associated species, while WT-CH mice exhibited increased abundance of TTPKO-associated species. These results demonstrate that co-housing facilitates microbial exchange, leading to convergence of gut microbiota.

### Microbiota transfer bidirectionally modulates systemic inflammation and pathology

**Fig. 2:**
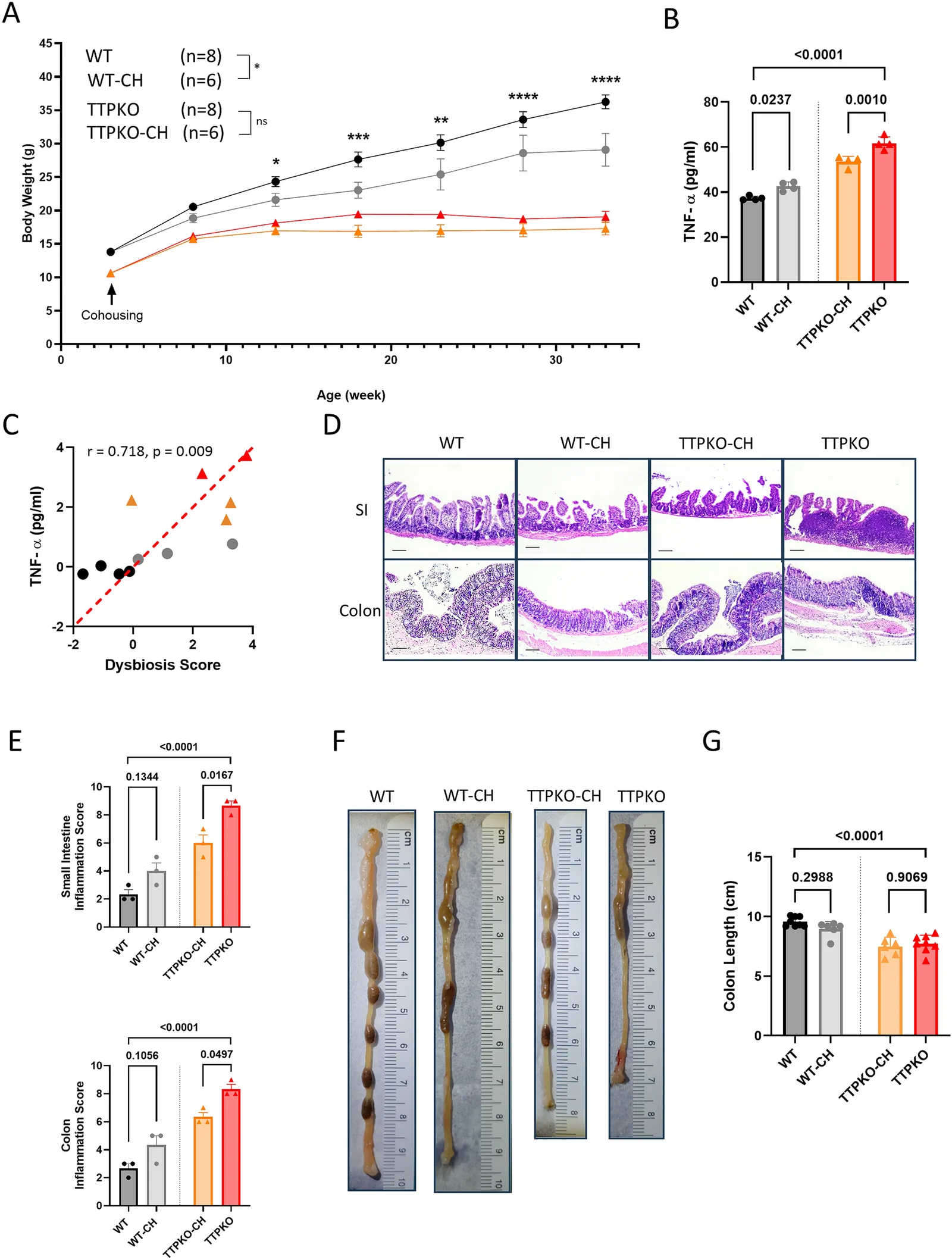
Microbiota transfer bidirectionally modulates systemic inflammation and pathology. **A** Body weight progression of all four groups of mice from 3 to 33 weeks of age. (n = 8 for WT, n = 6 for WT-CH, n = 8 for TTPKO, n = 6 for TTPKO-CH). **B** Serum TNF-α levels measured by ELISA at 33 weeks (*n* = 4 per group). **(C)** Correlation analysis between the microbial dysbiosis score and serum TNF-α levels at 33 weeks. **D** Representative histological H&E images of the small intestine (SI) and colon. **E** Histological inflammation scores for the small intestine and colon (n = 3 per group). **F** Representative gross images of the colon from each group at 33 weeks. **G** Colon length measurements at 33 weeks (n = 8 for WT, n = 6 for WT-CH, n = 8 for TTPKO, n = 6 for TTPKO-CH). Statistical significance was determined by two-way ANOVA with Tukey’s post-hoc test for body weight and one-way ANOVA with Tukey’s post-hoc test for bar graphs. Data are represented as mean ± SEM

Next, we investigated whether the microbiota transfer could modulate the host’s inflammatory phenotype. As previously established [11], TTPKO mice exhibited severe growth retardation. Co-housing triggered a bidirectional effect on body weight; WT-CH mice displayed significant weight gain retardation beginning at 13 weeks, partially adopting the TTPKO phenotype, whereas TTPKO-CH mice showed no significant weight recovery (Fig. 2A).

This bidirectional modulation was more pronounced in systemic inflammatory markers. Serum concentrations of the pro-inflammatory cytokine TNF-α were significantly increased in WT-CH mice compared to control WT mice. In contrast, TTPKO-CH mice, which received microbiota from WT mice, displayed a significant reduction in TNF-α levels (Fig. 2B). Correlation analysis revealed a significant positive correlation between the microbial dysbiosis score and serum TNF-α concentrations across all groups (Fig. 2C).

This inflammatory shift was also evident at the intestinal tissue level. Histological analysis revealed a trend towards higher inflammation scores in the small intestine and colon of WT-CH mice compared to WT mice. Conversely, TTPKO-CH mice showed a significant reduction in inflammation scores relative to TTPKO mice, confirming that gut inflammation is bidirectionally modulated under SPF conditions (Fig. 2D-E). Macroscopically, WT-CH mice showed a tendency toward shorter colon length compared to WT mice, while TTPKO-CH mice did not recover the shortened colon phenotype (Fig. 2F-G).

To clarify the specific requirement of the microbial community for these phenotypic shifts, we evaluated the germ-free (GF) cohort. Strikingly, the transmission of the inflammatory phenotype to healthy mice was completely abrogated in the absence of microbes; GF WT-CH mice showed no increase in serum TNF-α or intestinal inflammation scores compared to GF WT mice (Supplementary Fig. 4). Interestingly, we observed a reduction in gut inflammation scores in GF TTPKO-CH mice compared to GF TTPKO mice. This distinction indicates that while the transmission of systemic inflammation to a healthy host is strictly microbiota-dependent, the amelioration of localized gut inflammation in diseased hosts may also involve microbiota-independent environmental or behavioral factors associated with co-housing. Furthermore, GF TTPKO mice still showed lower body weights compared to GF WT mice, reflecting the intrinsic effect of the TTP gene deficiency.

### Dysbiotic microbiota drives the systemic expansion of osteoclastogenic monocytic myeloid-derived suppressor cells

**Fig. 3:**
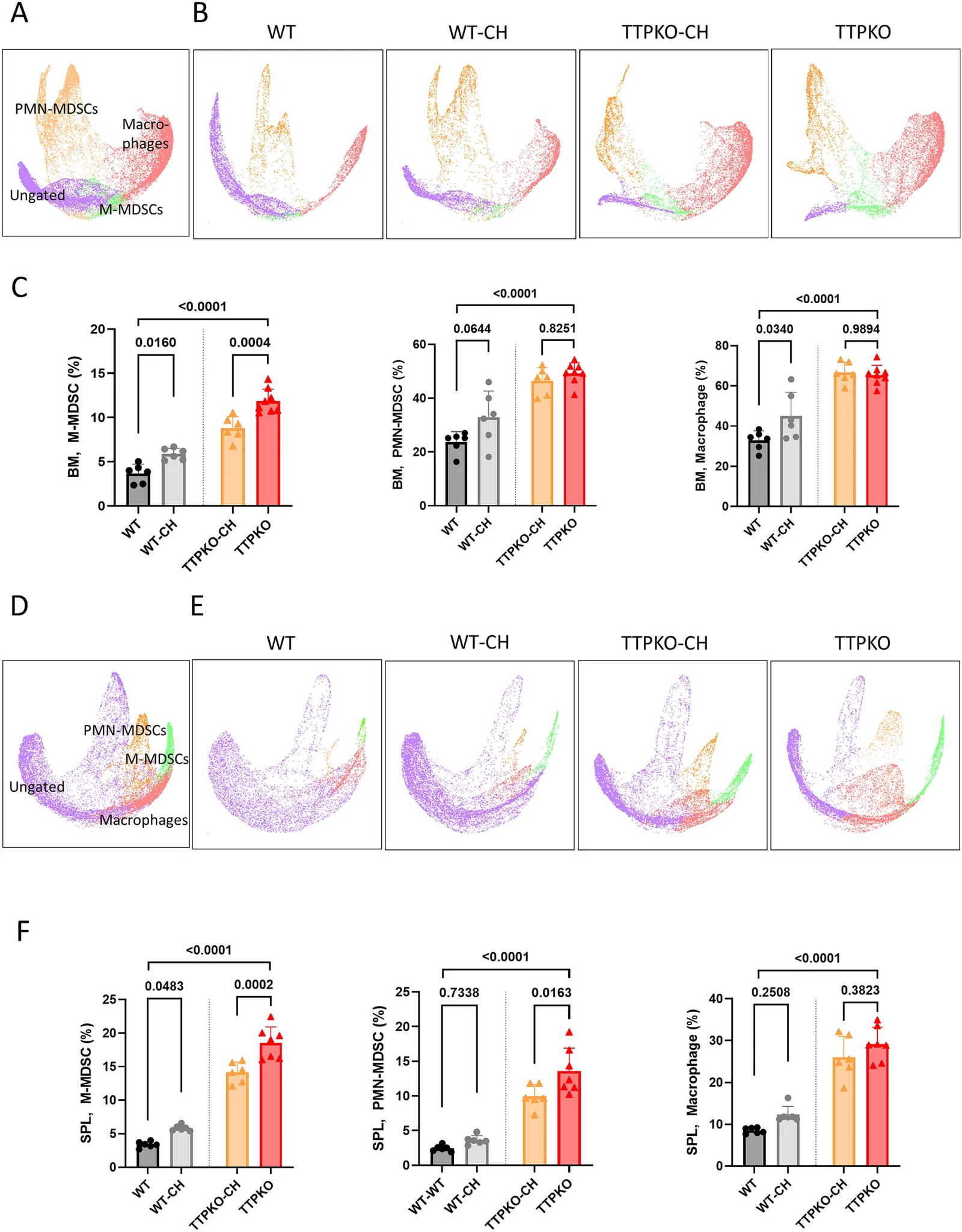
Gut microbiota drives the systemic expansion of M-MDSCs in SPF mice. **A** PHATE plot visualizing myeloid cell populations in the bone marrow, including monocytic myeloid-derived suppressor cells (M-MDSCs; CD11b^+^ Ly6C^high^ Ly6G^−^), polymorphonuclear MDSCs (PMN-MDSCs; CD11b^+^ Ly6C^low^ Ly6G^+^), and macrophages (CD11b^+^ F4/80^+^). **B** Representative PHATE plots for bone marrow from each experimental group. **C** Quantification of the percentage of M-MDSCs, PMN-MDSCs, and macrophages (F4/80^+^) within the CD11b^+^ cell population in the bone marrow. **D** PHATE plot visualizing myeloid cell populations in the spleen. **E** Representative PHATE plots for spleen from each experimental group. **F** Quantification of the percentage of M-MDSCs, PMN-MDSCs, and macrophages within the CD11b^+^ cell population in the spleen. Statistical significance was determined by one-way ANOVA with Tukey’s post-hoc test. Data are represented as mean ± SEM (n = 6 for WT, n = 6 for WT-CH, n = 6 for TTPKO-CH, n = 8 for TTPKO)

To confirm the cellular mechanisms driving this systemic inflammation, we analyzed myeloid cell populations in the bone marrow (BM) and spleen (SPL) using an established flow cytometry gating strategy (Supplementary Fig. 5). In the SPF setting, analysis revealed that TTPKO mice had a dramatic expansion of monocytic myeloid-derived suppressor cells (M-MDSCs) compared to WT mice (Fig. 3). This osteoclastogenic M-MDSC expansion was directly and bidirectionally modulated by co-housing. WT-CH mice showed a significant increase in M-MDSCs in both the BM and SPL, while TTPKO-CH mice showed a significant reduction, mirroring the changes in TNF-α levels (Fig. 3C, 3 F). While polymorphonuclear MDSCs (PMN-MDSCs) and macrophages were also expanded in TTPKO mice, their responses to co-housing were more complex. The increase in BM macrophages was partially transmissible, whereas the expansion of PMN-MDSCs was not transmitted to WT-CH mice (Fig. 3C, 3 F).

**Fig. 4:**
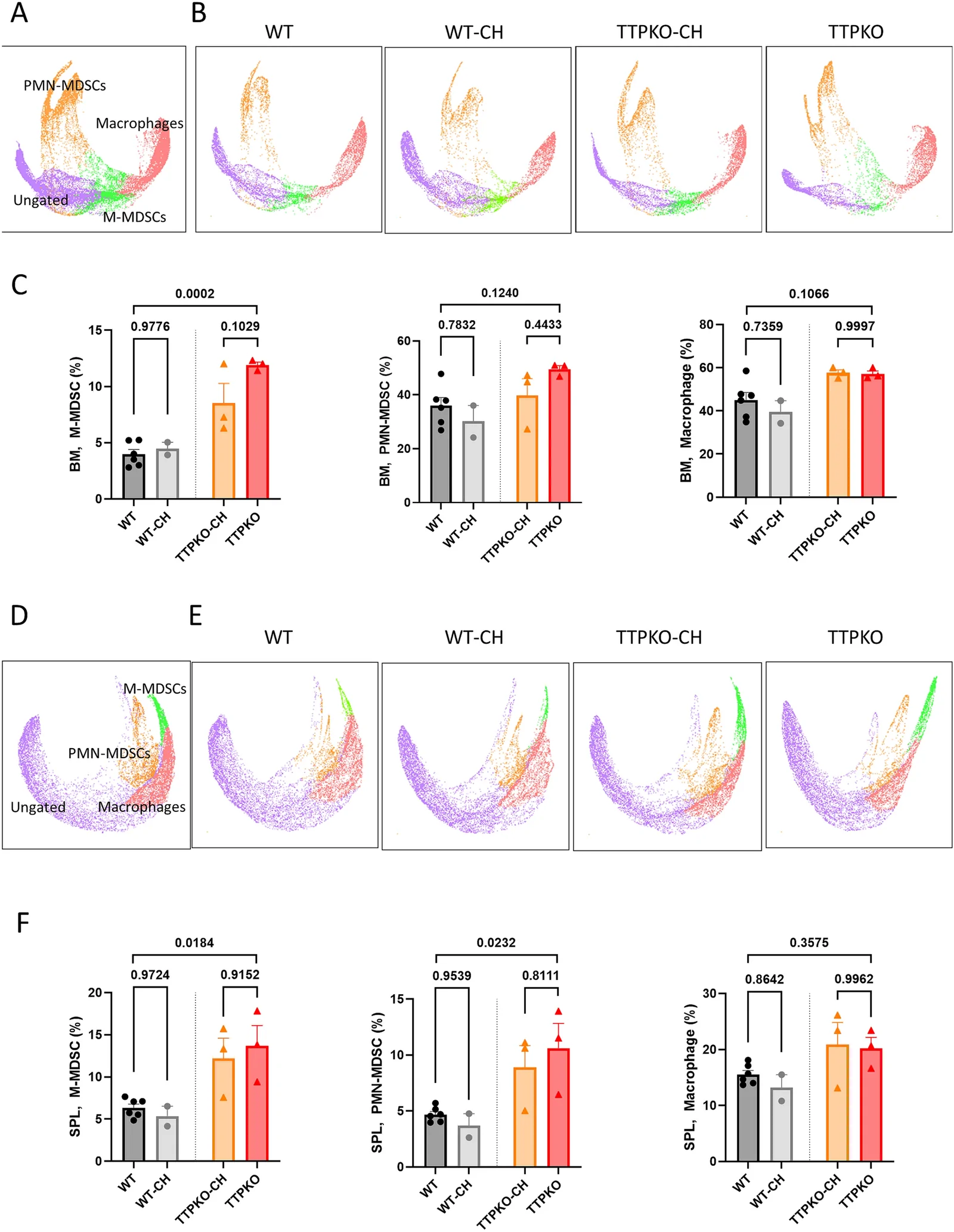
Expansion of myeloid cells is abrogated in a germ-free environment. Myeloid cell populations were analyzed in germ-free mice at 33 weeks. **A** Representative PHATE plots visualizing myeloid cell populations in the bone marrow. **B** Representative PHATE plots for bone marrow from each experimental group of germ-free mice. **C** Quantification of the percentage of M-MDSCs, PMN-MDSCs, and macrophages in the bone marrow of germ-free mice, showing no significant differences between groups. **D** Representative PHATE plots visualizing myeloid cell populations in the spleen. **E** Representative PHATE plots for spleen from each experimental group of germ-free mice. **F** Quantification of the percentage of M-MDSCs, PMN-MDSCs, and macrophages in the spleen of germ-free mice, confirming the absence of microbiota-driven expansion. Statistical significance was determined by one-way ANOVA. Data are represented as mean ± SEM (n = 6 for WT, n = 2 for WT-CH, n = 3 for TTPKO-CH, n = 3 for TTPKO)

Crucially, these co-housing-induced cellular alterations were entirely prevented in the GF environment. In the absence of microbial communities, no significant differences were observed in M-MDSCs or other myeloid populations in either the BM or SPL among any of the GF groups (Fig. 4). Together, these findings demonstrate that the gut microbiota is the essential driver for the systemic expansion and transmission of osteoclastogenic M-MDSCs.

### Microbiota-driven inflammation deteriorates trabecular bone microarchitecture

**Fig. 5:**
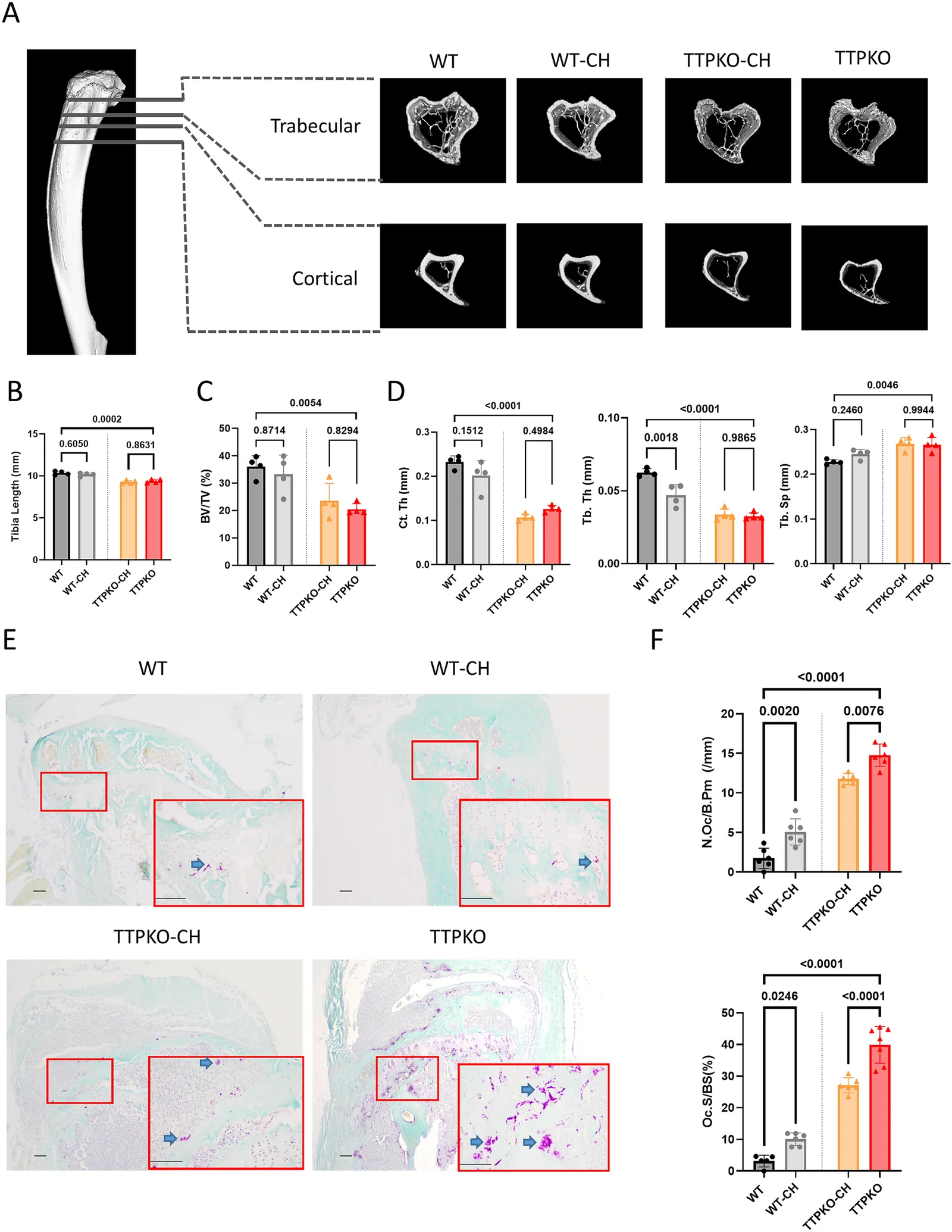
Microbiota-driven inflammation promotes a transmissible bone loss phenotype in SPF mice. **A** Representative micro-computed tomography (µCT) images of the proximal tibia showing trabecular and cortical bone architecture from each group at 33 weeks. **B** Tibia length measurements for all groups. **C** Quantification of bone volume fraction (BV/TV) from µCT analysis. **D** Quantification of cortical bone thickness (Ct.Th), trabecular bone thickness (Tb.Th), and trabecular bone separation (Tb.Sp) from µCT analysis. **E** Representative TRAP-stained images of the proximal tibia. The main images were taken at 4X magnification (Scale bar = 100 µm), and the insets indicated by red boxes show higher magnification views at 10X (Scale bar = 100 µm). Blue arrows indicate TRAP-positive multinucleated osteoclasts (stained in purplish-red). **F** Histomorphometric quantification of the number of TRAP-positive osteoclasts per bone perimeter (N.Oc/B.Pm) and osteoclast surface per bone surface (Oc.S/BS). Statistical significance was determined by one-way ANOVA with Tukey’s post-hoc test. Data are represented as mean ± SEM (n = 4 for WT, n = 4 for WT-CH, n = 4 for TTPKO-CH, n = 4 for TTPKO)

Given that M-MDSCs serve as osteoclast precursors, we investigated whether the microbiota-driven inflammation impacted bone homeostasis. Micro-computed tomography (µCT) analysis in SPF mice revealed a severe bone loss phenotype in TTPKO mice, characterized by significantly reduced bone volume fraction (BV/TV), cortical bone thickness (Ct.Th) and trabecular bone thickness (Tb.Th), along with increased trabecular bone separation (Tb.Sp) (Fig. 5A, 5 C, 5D).

We next assessed if this pathological bone phenotype was transmissible. Interestingly, while co-housing in WT-CH mice did not lead to significant changes in tibia length or overall bone volume (Fig. 5B-C), we observed a specific and highly significant alteration in the trabecular bone microarchitecture. WT-CH mice had significantly reduced trabecular bone thickness (Tb.Th) compared to WT mice, demonstrating that a discrete feature of bone quality – trabecular deterioration – was transmissible (Fig. 5D). Consistent with earlier phenotypic markers, co-housing did not rescue the established structural bone defects in TTPKO-CH mice.

To directly assess whether this altered trabecular microarchitecture was driven by enhanced osteoclast function, we performed TRAP staining on the tibia sections. Consistent with the systemic expansion of M-MDSCs, WT-CH mice exhibited a significant increase in both the number of TRAP-positive osteoclasts per bone perimeter (N.Oc/B.Pm) and osteoclast surface area (Oc.S/BS) compared to WT controls (Fig. 5E, 5 F). This provides direct functional evidence that the transmissible dysbiotic microbiota stimulates in vivo osteoclastogenesis.

### Transmissible trabecular bone loss is microbiota-dependent

**Fig. 6:**
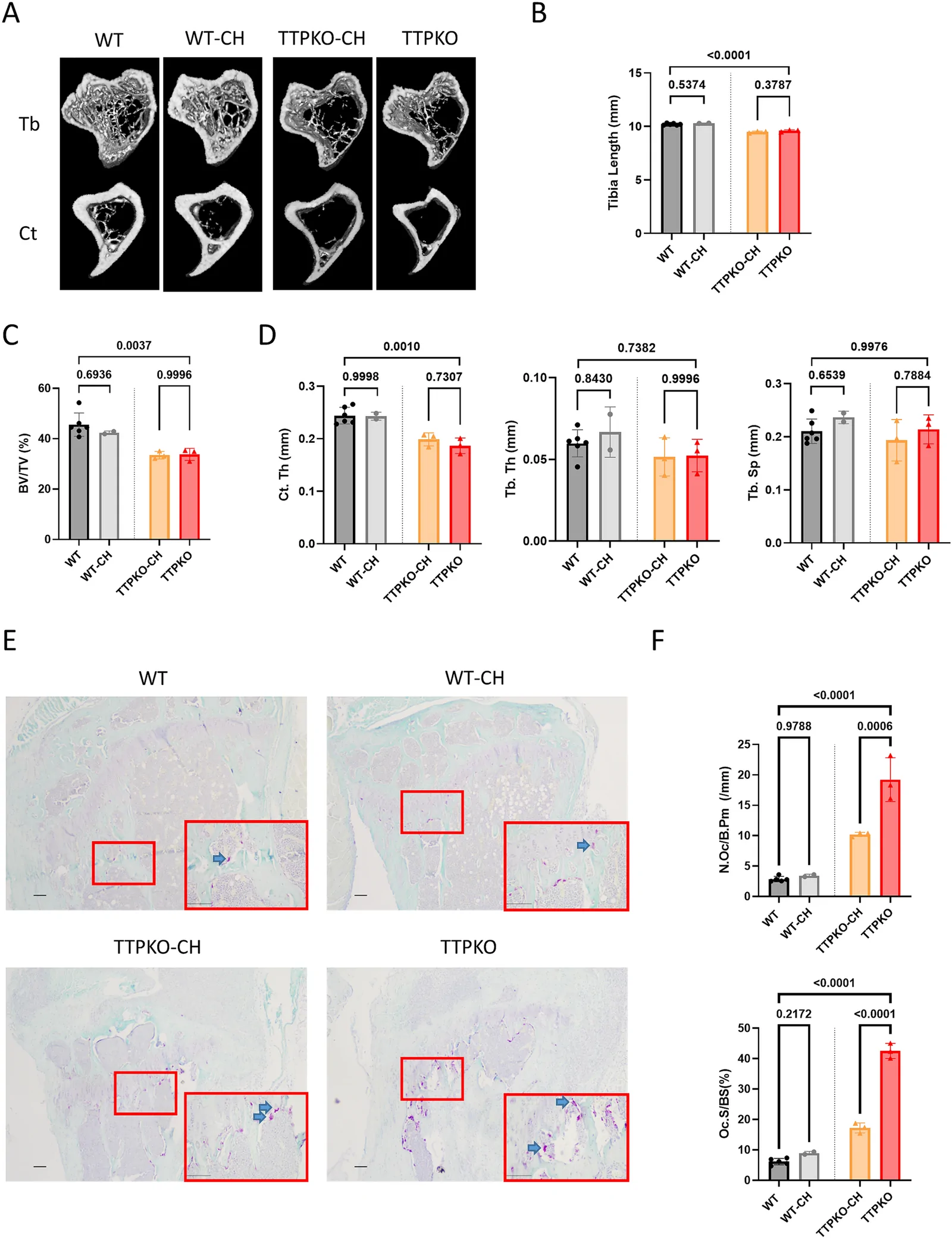
Transmissible trabecular bone loss is microbiota-dependent and abrogated in germ-free mice. Bone phenotype was analyzed in germ-free mice at 33 weeks. **A** Representative µCT images of the proximal tibia from each germ-free group. **B** Tibia length measurements for all germ-free group. **C** Quantification of bone volume fraction (BV/TV) from µCT analysis. **D** Quantification of cortical bone thickness (Ct.Th), trabecular bone thickness (Tb.Th), and trabecular bone separation (Tb.Sp) from µCT analysis. **E** Representative TRAP-stained images of the proximal tibia in germ-free mice. The main images are shown at 4X magnification (Scale bar = 100 µm), with corresponding 10X magnified insets (Scale bar = 100 µm) of the regions marked by red boxes. Blue arrows indicate TRAP-positive multinucleated osteoclasts. **F** Histomorphometric quantification of N.Oc/B.Pm and Oc.S/BS in germ-free mice. The results show that co-housing-induced bone loss is abrogated, though a baseline bone deficit persists in TTPKO mice compared to WT mice. Statistical significance was determined by one-way ANOVA with Tukey’s post-hoc test. Data are represented as mean ± SEM (n = 6 for WT, n = 2 for WT-CH, n = 3 for TTPKO-CH, n = 3 for TTPKO)

Finally, to further test that the transmissible trabecular deterioration was driven by the microbiome, we analyzed bone phenotypes in GF mice. In the absence of microbiota, the co-housing-induced deterioration of trabecular bone (Tb.Th) observed in WT-CH mice was completely prevented (Fig. 6). Interestingly, GF TTPKO mice still retained a baseline bone deficit (lower BV/TV and Ct.Th) compared to GF WT mice (Fig. 6C, 6D). This effectively dissociates the pathology into two distinct components: a baseline genetic bone deficit caused directly by TTP deficiency, and a superimposed, transmissible microbiota-driven pathology that specifically exacerbates the deterioration of trabecular microarchitecture.

In line with the structural preservation, TRAP staining of GF bone tissues revealed that the co-housing-induced osteoclast activation was entirely abolished in the absence of microbes. GF WT-CH mice showed no significant increases in N.Oc/B.Pm or Oc.S/BS relative to GF WT mice (Fig. 6E, 6 F), confirming that the enhanced osteoclastogenesis is strictly microbiota-dependent.

## Discussion

In this study, we delineated a self-reinforcing feedback loop between host genetics, the gut microbiota, and skeletal homeostasis. Our central hypothesis was that the dysbiotic microbiota arising in TTP-deficient mice is not simply a downstream reflection of chronic inflammation but instead acts as a transmissible driver that amplifies bone pathology. Using co-housing and germ-free models, we demonstrated that TTP deficiency initiates microbial imbalance, and this dysbiotic community heightens systemic inflammation, which further entrenches dysbiosis. This vicious cycle promotes the systemic expansion of osteoclastogenic M-MDSCs and accelerated trabecular bone deterioration, establishing that the gut microbiome functions as a central amplifier and transmitter of TTP-mediated skeletal disease.

A key finding of this study is the bidirectional modulation of the inflammatory and skeletal phenotypes through co-housing. Transfer of a “healthy” microbiota from WT mice into TTPKO mice (TTPKO-CH) partially restored microbial balance and significantly reduced circulating TNFα. In contrast, transfer of a “dysbiotic” microbiota from TTPKO mice into WT mice (WT-CH) was sufficient to induce hallmark features of the inflammatory state. These results demonstrate that the gut microbiota is a powerful determinant of systemic inflammatory tone. Moreover, we identified a direct link between microbial status and the systemic expansion of M-MDSCs. Crucially, this cellular phenotype—the expansion of osteoclast precursors and subsequent in vivo osteoclast activation—was transmissible between co-housed mice and, importantly, completely absent under germ-free conditions. Together, these findings establish the gut microbiota as the essential upstream driver of osteoclastogenic M-MDSC expansion and functional bone resorption, serving as a central mediator of the inflammatory bone loss observed in TTP deficiency.

Notably, however, this bidirectional modulation was not symmetric at the level of skeletal outcomes. While transfer of the TTPKO-associated dysbiotic microbiota to healthy WT mice induced trabecular microarchitectural degradation, the reciprocal transfer of WT microbiota to TTPKO mice did not rescue the established skeletal deficit. We propose this reflects the temporal dynamics of bone loss relative to the window of microbiome intervention. In C57BL/6 mice, trabecular bone volume peaks as early as 6–8 weeks of age and declines progressively thereafter [21, 22], and TTPKO mice develop osteoclastogenic inflammation coincident with this critical period of skeletal accrual [11]. Substantial trabecular architectural deterioration is therefore likely established before microbial convergence via co-housing can meaningfully attenuate bone resorption. This is consistent with the broader microbiome-bone literature, in which microbiome-targeted interventions have demonstrated efficacy primarily in the prevention of bone loss rather than reversal of established deficits [23, 24]. Future studies employing earlier microbiome intervention, initiated prior to the onset of the inflammatory phenotype, will be essential to determine whether restoration of microbial homeostasis can preserve trabecular architecture in genetically susceptible hosts.

Our experiments in the GF model further distinguished two separate phenomena: a baseline, microbiota-independent bone deficit intrinsic to host TTP deficiency, and a second, transmissible layer of pathology driven by the microbiota. Interestingly, under GF co-housing conditions, TTP-deficient mice showed a slight trend toward reduced localized gut inflammation. This suggests that although the gut microbiota is the principal driver of the transmissible inflammatory phenotype, microbiota-independent environmental factors associated with co-housing—such as altered behavioral stress, thermoregulation, or the exchange of sterile dietary metabolites through coprophagia—may exert modest immunomodulatory effects that partially alleviate localized inflammation [25, 26]. Importantly, transmission of systemic inflammation and trabecular deterioration to healthy WT mice was completely abolished in the GF environment, consistent with a microbiome-dependent spread of the disease phenotype. We acknowledge that the sample size of our GF cohort does not provide statistical insights due to inherent logistical challenges of generating and maintaining aged gnotobiotic co-housing models; however, the complete abrogation of the transmissible phenotypes provides robust proof-of-concept supportive data.

A notable finding from our study was the significant increase in histological gut inflammation scores in TTPKO mice under steady-state conditions. This observation diverges from some original studies where TTP-deficient mice did not exhibit overt spontaneous colitis [8, 27]. We propose that factors such as facility-specific microbiota differences [25, 26, 28], the older age of mice in our longitudinal analysis, and methodological variations in scoring [25, 26, 28] may account for this difference. Thus, our findings suggest that under certain microbial and temporal conditions, TTP deficiency can manifest as spontaneous gut inflammation, which may further fuel the systemic osteoclastogenic drive.

Our finding that a dysbiotic microbiota can selectively compromise bone quality offers new mechanistic insight into the gut-bone axis. Previous work showed that gut microbiome perturbations can weaken bone tissue-level strength without significantly affecting bone mass. Consistent with this, our model showed that the transmissible phenotype in WT-CH mice was defined by a specific loss of trabecular thickness—a critical parameter of bone microarchitecture—rather than a gross loss of bone mass [29]. This suggests that the dysbiotic community from TTPKO mice drives pathology primarily by impairing bone matrix quality. Potential mechanisms include elevated systemic inflammatory mediators (e.g., LPS) [30, 31], alterations in microbial-derived metabolites like Vitamin K or short-chain fatty acids (SCFAs) [32–34], and the direct action of expanded osteoclast precursors (M-MDSCs). The fact that distinct microbial signatures can drive discrete aspects of skeletal pathology reinforces that it is the specific TTP-associated dysbiosis, not just dysbiosis in general, that acts as the pathogenic driver.

Critically, our TRAP staining results provide direct functional evidence linking the microbiota-driven systemic M-MDSC expansion to pathogenic osteoclastogenesis in the bone microenvironment. While previous studies implicated M-MDSCs as potential osteoclast precursors, our current data definitively demonstrate that microbiota-driven resorptive activity directly accounts for the selective deterioration of trabecular bone observed in WT-CH mice. Furthermore, our results demonstrate a systemic skeletal impact of the gut microbiome, in contrast to the site-specific effects often seen with oral microbiota [35]. This suggests that the dysbiotic gut microbiome influences bone homeostasis via the circulatory system, distributing inflammatory mediators and osteoclast precursors throughout the body [36, 37].

While our TRAP staining data clearly demonstrate that microbiota-driven trabecular thinning involves increased local osteoclastogenesis, we acknowledge that the absence of dynamic histomorphometry and systemic serum bone turnover markers (e.g., CTX-1, P1NP) represents a limitation of the present study. Consequently, although enhanced osteoclastic bone resorption is a confirmed driver, we cannot rule out the possibility of concurrent suppression in osteoblastic bone formation. Furthermore, although systemic M-MDSC expansion corresponds with pathogenic osteoclastogenesis, the lack of direct M-MDSC depletion or adoptive transfer experiments in this study means we cannot completely rule out the contribution of other concurrent immunoregulatory pathways. Future studies incorporating calcein/alizarin double-labeling and biochemical indices of bone remodeling activity will be essential to fully define the in vivo kinetics of the microbiota-driven skeletal phenotype described here.

The clinical implications of our findings address the fundamental role of systemic chronic inflammation (SCI) in age-related bone loss. SCI is a common etiological driver linking conditions like cardiovascular disease and osteoporosis. Our results, demonstrating that a transmissible dysbiotic microbiota acts as a potent amplifier of genetic predisposition, offer a unifying explanation for the clinical heterogeneity seen in osteoporotic patients. This suggests that the gut microbiome is a modifiable factor that influences the severity of inflammatory bone diseases. Therefore, interventions aiming to restore microbial homeostasis—such as probiotics or fecal microbiota transplantation—could represent a promising strategy to dampen osteoclastogenic inflammation and mitigate bone fragility, even when the underlying genetic cause cannot be altered.

In conclusion, TTP deficiency fosters a dysbiotic gut microbiota that amplifies systemic inflammation, expands pathogenic osteoclast precursors (M-MDSCs), and drives trabecular bone loss. These findings identify the microbiome as a transmissible determinant of skeletal disease severity and a potential therapeutic target in genetically susceptible individuals.

## Supplementary Information

Below is the link to the electronic supplementary material.

Supplementary file1 (TIF 2100 KB). Figure 1: LEfSe analysis identifies key bacterial taxa differentiating WT and TTPKO groups. Linear discriminant analysis effect size (LEfSe) plots showing bacterial taxa differentially abundant between WT and TTPKO at week 33. An LDA score ≥ 2 was used as the cutoff. Bar length represents the LDA score, indicating the effect size of each taxon as a biomarker for its respective group. Taxa are colored according to the group in which they are most abundant

Supplementary file2 (TIF 1619 KB). Figure 2: LEfSe analysis identifies key bacterial taxa differentiating the housing groups. LEfSe plots showing bacterial taxa differentially abundant among the four housing groups at week 33. An LDA score ≥ 2 was used as the cutoff. Bar length represents the LDA score, indicating the effect size of each taxon as a biomarker for its respective group. Taxa are colored according to the group in which they are most abundant

Supplementary file3 (TIF 2473 KB). Figure 3: Distribution of WT- and TTPKO-associated OTUs across experimental groups. Heatmap showing the centered log-ratio (CLR) abundance of operational taxonomic units (OTUs) normalized to Z-scores at week 33. OTUs were identified as differentially abundant based on Linear discriminant analysis effect size (LEfSe) analysis with an LDA score ≥ 2 comparing WT and TTPKO groups. The color bar indicates the enrichment of each OTU in WT-CH vs. WT, TTPKO vs. TTPKO-CH, and WT vs. TTPKO, respectively, as determined by LEfSe

Supplementary file4 (TIF 2129 KB). Figure 4: Transmission of systemic inflammatory phenotypes is abrogated in germ-free mice. Phenotypic analysis of germ-free mice at 33 weeks. Bar graphs show (A) body weight, (B) colon length, (C) serum TNF-α levels, and (D) histological inflammation scores for the small intestine and colon. In the absence of microbiota, the co-housing-induced transmission of systemic inflammation (e.g., increased TNF-α) to WT mice was completely prevented. Notably, a partial amelioration of localized gut inflammation was observed in GF TTPKO-CH mice, highlighting that while disease transmission is microbiota-dependent, localized amelioration may involve microbiota-independent factors. Statistical significance was determined by one-way ANOVA. Data are represented as mean ± SEM (n=6 for WT, n=2 for WT-CH, n=3 for TTPKO-CH, n=3 for TTPKO)

Supplementary file5 (TIF 1611 KB). Figure 5: Flow cytometry gating strategy for myeloid cell analysis. Representative plots showing the hierarchical gating strategy used to identify myeloid cell populations from bone marrow and spleen. The strategy involved initial gating on single cells (FSC-H vs FSC-A), followed by a viability gate to exclude dead cells. From the viable population, CD11b+ cells were selected. These were further resolved based on Ly6C and Ly6G expression to identify M-MDSCs (CD11b+Ly6ChighLy6G-) and PMN-MDSCs (CD11b+Ly6ClowLy6G+). Macrophages were identified as CD11b+F4/80+

## Data Availability

The 16S data generated in this study is available in the Sequence Reads Archive (Accession number PRJNA1305457). Additional datasets generated and/or analyzed during the current study are available from the corresponding author on reasonable request.
